# Oxygen Bridge Governs OER via Deep Self-Reconstruction in Fe–Co Oxyhydroxides

**DOI:** 10.3390/molecules31010096

**Published:** 2025-12-25

**Authors:** Mingyu Liu, Bowen Pei, Hongyu Ba, Wei Ni, Huaheng Zhao, Shuang Chen, Jiamin Zhao, Jinsheng Zhao

**Affiliations:** 1 College of Chemistry and Chemical Engineering, China University of Petroleum (East China), Qingdao 266580, China; liumingyu991204@163.com (M.L.); peibowen2024@163.com (B.P.); 17669793559@163.com (H.B.); nw17585134644@163.com (W.N.); 15212531621@163.com (H.Z.); 2 School of Chemistry and Chemical Engineering, Liaocheng University, Liaocheng 252059, China

**Keywords:** oxygen evolution reaction, Fe–Co oxyhydroxides, deep self-reconstruction, electrocatalysis

## Abstract

The oxygen evolution reaction (OER) in water splitting involves complex multi-electron–proton transfer processes and represents the rate-determining step limiting overall electrolysis efficiency. Developing non-noble-metal catalysts with high activity and stability is therefore essential. Herein, a heterogeneous synthesis strategy was employed to in situ construct an iron-rich layered sulfate precursor (Fe_0.42_Co_0.58_-SO_4_/NF) on nickel foam, which underwent deep self-reconstruction in alkaline electrolyte to form nanoflower-like Fe_0.42_Co_0.58_OOH/NF. The optimized catalyst maintained its iron-rich composition and hierarchical structure, delivering outstanding OER performance with an overpotential of 220 mV at 10 mA·cm^−2^, a Tafel slope of 31.9 mV·dec^−1^, and stability exceeding 12 h at 600 mA·cm^−2^. Synchrotron analyses revealed dynamic transitions between mono-μ-O and di-μ-O Fe–M (M = Fe, Co) oxygen bridges during reconstruction, which enhanced both structural robustness and active-site density. The Fe-rich environment promoted the formation of Fe^3+^–O–Fe^3+^ units that synergized with Co^4+^ species to activate the lattice oxygen mechanism (LOM), thereby accelerating OER kinetics. This work elucidates the key role of oxygen-bridge geometry in optimizing catalytic activity and durability, providing valuable insights into the rational design of Fe–Co-based non-noble-metal catalysts with high iron content for efficient water oxidation.

## 1. Introduction

Hydrogen production via water splitting is one of the key pathways toward establishing a sustainable hydrogen energy system [1]. Among the half-reactions involved, the oxygen evolution reaction (OER) occurs at the anode and often serves as the bottleneck limiting the overall energy efficiency of water splitting, owing to its complex multi-step electron–proton transfer process and the high energy barrier for O–O bond formation [2]. Developing efficient, stable, and cost-effective electrocatalysts therefore remains a central challenge for improving OER performance. At present, although noble metal oxides such as IrO_2_ and RuO_2_ exhibit excellent catalytic activity, their high cost and limited availability severely restrict large-scale commercial applications [3].

Iron-group (Fe, Co, Ni) based oxides and layered double hydroxides (LDHs) have attracted extensive attention due to their outstanding electrocatalytic performance toward the OER in alkaline media, among which FeCo LDH materials are particularly representative [4]. Studies have shown that the appropriate incorporation of Fe not only induces the reconstruction of the crystal structure in Co-based hydroxides [5], but also generates Fe–M (M = Co, Fe) active centers with μ-O (oxo-bridge) structures, thereby providing more highly active sites and significantly enhancing the intrinsic catalytic activity. However, excessive Fe content tends to reduce the electrical conductivity of the material and aggravate its dissolution under alkaline conditions, thus compromising stability. Conversely, higher Co content helps maintain structural integrity but often at the expense of catalytic activity [6,7]. Therefore, achieving a reproducible and scalable synergistic balance between activity and stability—while overcoming the Fe/Co molar ratio limitation (≤0.33) caused by the intrinsic electrostatic repulsion between Fe^3+^–O–Fe^3+^ pairs—remains one of the key challenges in the rational design of FeCo LDH catalysts [8].

Beyond compositional optimization, understanding structural self-reconstruction under operating potentials and identifying the true active phase are crucial for elucidating reaction mechanisms and guiding rational catalyst design [7]. In situ Raman and synchrotron X-ray absorption spectroscopy (XAS) studies have revealed that, under anodic polarization, LDH precursors undergo progressive deintercalation or substitution of interlayer anions (e.g., SO_4_^2−^, NO_3_^−^, Cl^−^), transforming into oxyhydroxide phases [8,9,10,11]. This phase evolution is often accompanied by lattice-oxygen activation, oxygen-vacancy formation, and dynamic reorganization of μ-oxo bridges, generating additional active sites. Notably, in situ XAS and electrochemical analyses have confirmed μ-oxo-bridged Fe–M (M = Co, Fe) units as key OER centers [12,13]. Ou et al. further demonstrated that adjacent Fe sites on transition metal (oxy)hydroxides act cooperatively during OER, where Fe-Fe (or Fe-M) coupling via bridging oxygen species facilitates access to high-valence Fe states and stabilizes critical reaction inter-mediates, leading to enhanced OER kinetics compared to isolated Fe sites [14]. Simultaneously, potential-driven Co^3+^ → Co^4+^ oxidation is accompanied by μ-oxo rearrangements, while Fe–Co μ-oxo motifs—through the strong Lewis acidity of Fe^3+^—modulate the Co^3+^/Co^4+^ redox potential, facilitate lattice-oxygen participation, and accelerate OER dynamics [15]. Therefore, precisely identifying the reconstruction-derived active phase and μ-oxo configurations is fundamental to advancing mechanistic understanding and enabling the rational design of efficient OER catalysts.

Nevertheless, previous studies have shown that electrochemical reconstruction typically occurs only within a few nanometers of the surface, forming an ultrathin active layer that is challenging to unambiguously resolve using conventional characterization techniques [16,17]. This shallow reconstruction limits the identification of the true active phase and hinders the establishment of reliable structure–performance correlations. Recently, however, several studies have revealed that, under specific precursor compositions and operating conditions, deep self-reconstruction can be induced, leading to a complete transformation into a uniform new phase. Such a process not only overcomes the limitations of surface-confined reconstruction but also provides a unique opportunity to directly probe the intrinsic active phase and elucidate its dynamic evolution [18].

Against this background, we construct an Fe-rich layered sulfate precursor in situ (Fe_0.42_Co_0.58_-SO_4_) on nickel foam and subsequently induce deep self-reconstruction under alkaline galvanostatic/potentiostatic conditions to yield Fe_0.42_Co_0.58_OOH/NF while preserving the nanoflower morphology. In situ spectroscopic and electrochemical analyses reveal that, during reconstruction, Fe–M (M = Fe, Co) oxygen-bridge motifs dynamically interconvert between mono-μ-O and di-μ-O configurations, which stabilize the framework and substantially increase the number of accessible active sites. Remarkably, Raman spectra confirm the abundant formation of Fe^3+^–O–Fe^3+^ linkages under Fe-rich conditions, exceeding the commonly cited Fe/Co compositional threshold of ≤0.33. Further investigation demonstrates that these Fe-rich bridging motifs cooperate with potential-induced Co^4+^ species to activate the lattice oxygen mechanism (LOM), thereby accelerating OER kinetics. Overall, this study underscores the pivotal role of oxygen-bridge geometry in simultaneously enhancing catalytic activity and stability, offering a new strategy for the rational design of Fe-rich, FeCo-based non-noble OER catalysts.

## 2. Results and Discussion

### 2.1. Structural Characterization of the Precursor Fe_0.42_Co_0.58_-SO_4_/NF

**Figure 1:**
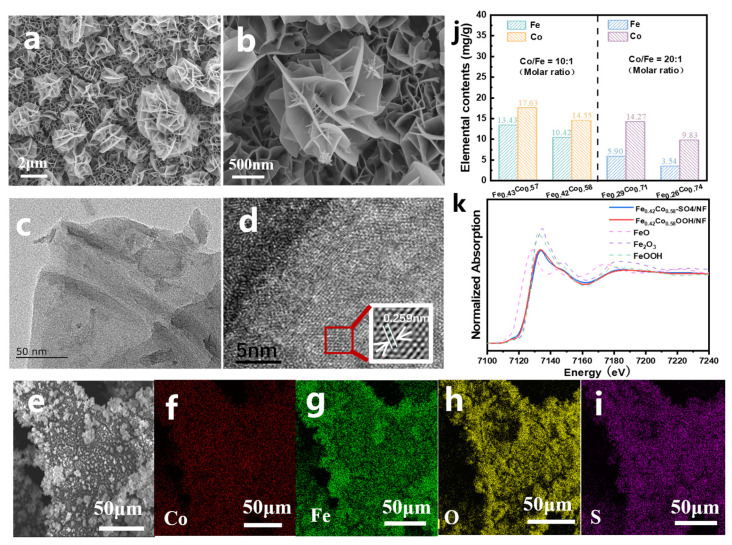
The precursor catalyst Fe_0.42_Co_0.58_-SO_4_/NF’s: (**a**,**b**) SEM image; (**c**) TEM image; (**d**) HRTEM image; (**e**–**i**) SEM-EDS elemental maps of Co, Fe, O, and S; (**j**) Variation in the content of Co and Fe before and after the OER process; (**k**) XANES spectra for Fe K-edge.

The precursor of Fe_0.42_Co_0.58_-SO_4_/NF was prepared by dissolving Co(NO_3_)_2_·6H_2_O and FeSO_4_·7H_2_O at a molar ratio of 10:1 in different solvents. Owing to the solubility difference between the two salts, Fe–Co nanoparticles were in situ-deposited onto the nickel foam (NF) surface, forming a nanoflower-like morphology. As observed in the SEM images (Figure 1a,b and Figure S1), the FeCo–SO_4_ nanosheets self-assembled into well-defined nanoflowers on NF. TEM analysis (Figure 1c) revealed that each nanoflower consisted of ultrathin nanosheet subunits, while the HRTEM image (Figure 1d and Figure S2) displayed lattice fringes with a spacing of 0.259 nm, corresponding to the (104) plane of layered double hydroxide (LDH) [19]. Furthermore, EDS mapping (Figure 1e–i, Table S1) confirmed the homogeneous distribution of Fe, Co, O, and S elements throughout the structure, with sulfur accounting for approximately 4 at%.

X-ray diffraction (XRD) analysis (Figure S3) of the precursor exhibited characteristic diffraction peaks in the 10–70° range. The broad peaks indicated relatively low crystallinity, and several reflections matched well with the standard card of poorly crystalline FeCo-LDH (PDF#00-050-0235). Notably, the FeCo-LDH pattern exhibited a low-angle (001) reflection at approximately 10°, while in Fe_0.42_Co_0.58_-SO_4_/NF, the presence of large interlayer SO_4_^2−^ ions resulted in a weaker diffraction peak at 8.1° [20]. Inductively coupled plasma atomic emission spectroscopy (ICP-AES, Figure 1j) further confirmed that the Co:Fe molar ratio in Fe_0.42_Co_0.58_-SO_4_/NF was approximately 3:2.

To further verify the chemical structure of the precursor, X-ray absorption fine structure spectroscopy (XAFS) was employed. The Fe K-edge X-ray absorption near-edge structure spectrum (XANES) of Fe_0.42_Co_0.58_-SO_4_/NF showed an absorption edge position close to that of Fe_2_O_3_, indicating that Fe predominantly existed in the +3 oxidation state (Figure 1k). The chemical state of cobalt was examined by X-ray photoelectron spectroscopy (XPS, Figure S4). The Co 2p spectrum exhibited clear spin–orbit splitting into Co 2p_3/2_ and Co 2p_1/2_ components at binding energies of 780.5 eV and 796.6 eV, respectively. Despite some peak overlap, qualitative analysis of the oxidation states was still possible. The Co 2p spectrum of Fe_0.42_Co_0.58_-SO_4_/NF indicated that cobalt predominantly existed as Co^2+^, as evidenced by the main peaks and the presence of shake-up satellite (“Sat”) peaks. Additional weak satellite features at 787.0 eV and 803.2 eV further confirmed the typical spectral characteristics of Co^2+^, consistent with previous reports [21,22,23].

### 2.2. Composition and Local Structure of Self-Reconstructed Fe_0.42_Co_0.58_OOH/NF

**Figure 2:**
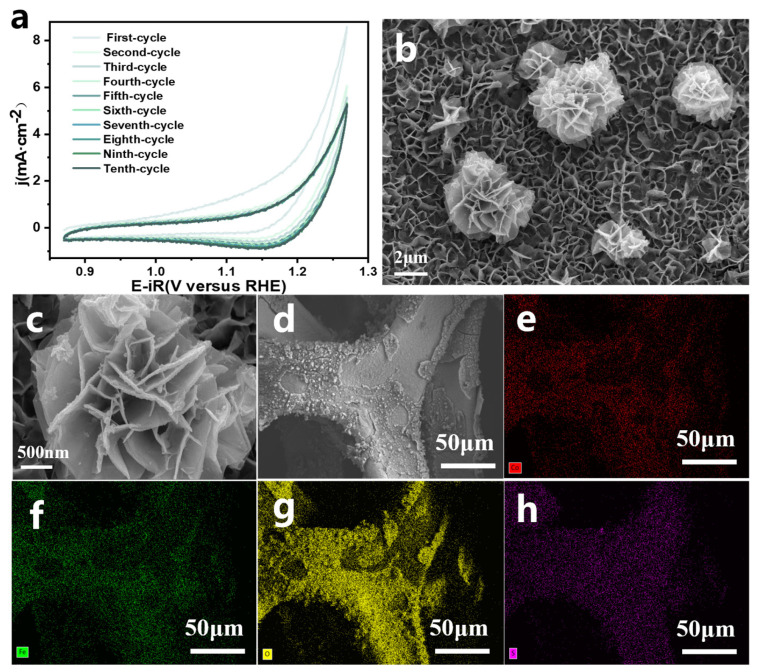
The working catalyst Fe_0.42_Co_0.58_OOH/NF’s: (**a**) Electrochemical activation by CV (10 cycles); (**b**,**c**) SEM image; (**d**–**h**) SEM-EDS elemental maps of Co, Fe, O, and S.

**Figure 3:**
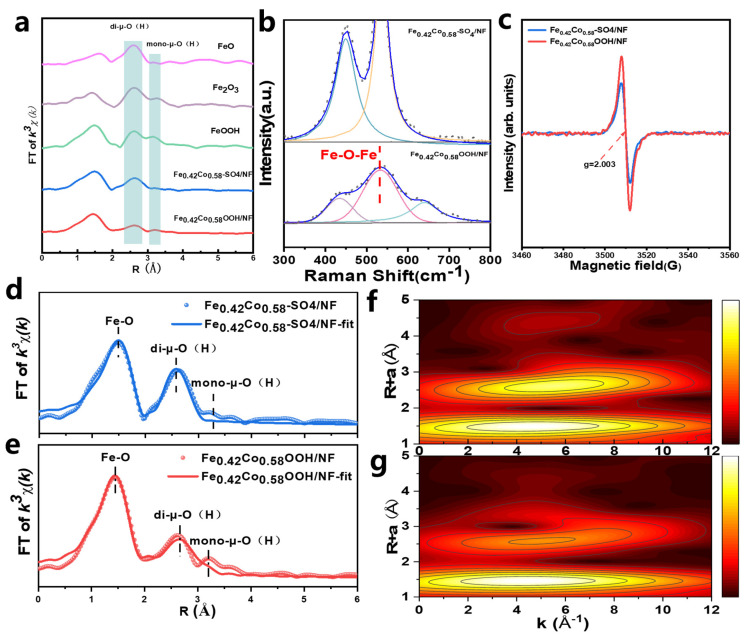
(**a**) EXAFS spectra for Fe K-edge; (**b**) Raman spectra of the Fe_0.42_Co_0.58_OOH/NF; (**c**) EPR spectra of Fe_0.42_Co_0.58_-SO_4_/NF and Fe_0.42_Co_0.58_OOH/NF recorded; (**d**) Fe_0.42_Co_0.58_-SO_4_/NF’s FT k^2^χ(R) EXAFS spectra for Fe K-edge; (**e**) Fe_0.42_Co_0.58_OOH/NF’s FT k^2^χ(R) EXAFS spectra for Fe K-edge; (**f**) Fe_0.42_Co_0.58_-SO_4_/NF’s WT transformed EXAFS spectra for Fe K-edge; (**g**) Fe_0.42_Co_0.58_OOH/NF’s WT transformed EXAFS spectra for Fe K-edge.

The precursor Fe_0.42_Co_0.58_-SO_4_/NF was electro-oxidized in 1 M KOH within a potential range of 0.87–1.27 V (vs. RHE). As shown in the cyclic voltammetry (CV) curves (Figure 2a), the electrochemically induced surface reconstruction gradually stabilized after ten activation cycles. SEM images confirmed that the nanoflower-like morphology was well preserved throughout the process (Figure 2b,c and Figure S5). Meanwhile, SEM-EDS analysis revealed a drastic decrease in sulfur content to only 0.06%, suggesting substantial structural reconstruction accompanied by electron redistribution during the OER process (Figure 2d–h and Table S2). To further probe the local environment and oxidation state of Fe, Fe K-edge XAFS analysis was performed. The XANES of Fe_0.42_Co_0.58_OOH/NF exhibited an absorption edge position comparable to that of FeOOH (Figure 1k), indicating that Fe mainly existed in the +3 oxidation state. Consistently, the EXAFS spectrum also closely resembled that of FeOOH (Figure 3a), corroborating the formation of a Fe^3+^-dominated oxyhydroxide phase. Additionally, ICP–AES showed negligible leaching of Fe and Co, and the final Co/Fe molar ratio remained nearly identical to that of the precursor (Figure 3a), confirming the successful formation of Fe_0.42_Co_0.58_OOH/NF.

The local structure of Fe was further probed by Fe K-edge XAFS. Previous studies have shown that Fe–O–M (M = Fe, Co) linkages can involve not only stable di-μ-O (edge-sharing) bridges but also a fraction of mono-μ-O (corner-sharing) bridges formed during electrochemical self-reconstruction [12,24,25,26]. The di-μ-O bridges, originating from two shared corner oxygen atoms between MO_6_ octahedra, help maintain the lattice stability, whereas mono-μ-O bridges, connected by a single oxygen atom, more readily induce local coordination unsaturation and oxygen vacancy formation, thus facilitating lattice oxygen participation in OER. Their cooperative effect enables FeMOOH (M = Ni, Co) to achieve both structural robustness and high catalytic activity. The EXAFS spectrum exhibited a weak peak at 3.2 Å for FeOOH, Fe_2_O_3_, and Fe_0.42_Co_0.58_OOH/NF (Figure 3a), which was absent in FeO and the precursor Fe_0.42_Co_0.58_-SO_4_/NF, and was attributed to Fe–M linkages coordinated by mono-μ-O(H) [24].

Further structural refinement using Fourier-transformed EXAFS and fitting analysis (Figure 3d,e, Table S3) revealed good consistency between experimental and modeled spectra in both K-space and R-space (Figure S6), confirming the reliability of the fitted local structural model. Compared with the precursor, the Fe–O bond length in Fe_0.42_Co_0.58_OOH/NF shortened from 2.00 Å to 1.96 Å, reflecting enhanced M–O covalency under operating potentials. The coordination number increased from 5 to nearly 6, suggesting a more complete FeO_6_ octahedron. Meanwhile, the Fe–O–M distance increased from 3.06 Å to 3.25 Å with a decrease in coordination number from 6 to 5, implying local structural defects. This was further corroborated by electron paramagnetic resonance (EPR, Figure 3c), which showed an increased intensity of oxygen vacancy (V_o_) signal at g = 2.003 [27]. Another notable observation was that the Fe–M (2.8–3.0 Å) di-μ-O contribution weakened and split in the EXAFS fitting, while new signals appeared at 3.3–3.5 Å, corresponding to mono-μ-O(H) FeO_6_ units [24]. Wavelet transform EXAFS (WT-EXAFS, Figure 3f,g) showed that the precursor Fe_0.42_Co_0.58_-SO_4_/NF featured pronounced Fe–O first-shell coordination and concentrated metal second-shell signals, along with weak long-range features [28]. After reconstruction, Fe_0.42_Co_0.58_OOH/NF retained the Fe–O nearest-neighbor shell, while the second-shell metal-related peaks broadened and attenuated at higher R, consistent with anion deintercalation and deep self-reconstruction from LDH to OOH [29].

Previous studies have shown that strong electrostatic repulsion inhibits the formation of Fe^3+^–O–Fe^3+^ bridge units, typically limiting the Fe/Co ratio in Fe_x_Co_1−x_OOH to no more than 1/2 (0 ≤ x ≤ 0.33) [30]. Interestingly, the Fe_0.42_Co_0.58_OOH/NF synthesized in this work exceeded this conventional Fe content limit. Raman spectroscopy of the precursor revealed characteristic Co–O vibrational modes (A_1g_ and E_g_) (Figure 3b) [31,32]. Upon reconstruction, Fe_0.42_Co_0.58_OOH/NF displayed a new peak at ~533 cm^−1^, corresponding to Fe^3+^–O–Fe^3+^ bridging vibrations [24]. This observation confirms that the elevated Fe content facilitated the formation of abundant Fe^3+^–O–Fe^3+^ structural units in the reconstructed catalyst.

### 2.3. Oxygen Evolution Reaction Performance

**Figure 4:**
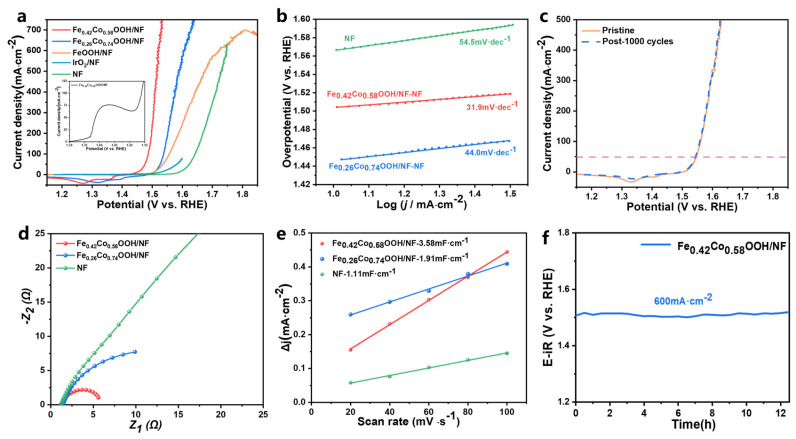
(**a**) LSV curves; (**b**) Tafel plots; (**c**) LSV curves of Fe_0.42_Co_0.58_OOH/NF before and after 1000 cycles CV durability test; (**d**) EIS plots; (**e**) C_dl_ plots derived from CV curve; (**f**) E-t curves of Fe_0.42_Co_0.58_OOH/NF at a current density of 600 mA cm^−2^.

To directly verify the occurrence of the oxygen evolution reaction, we employed in situ differential electrochemical mass spectrometry (DEMS) to monitor gas products in real time during the catalytic process (Figure S7). The experiment was conducted under an argon atmosphere, beginning with a 10-min baseline acquisition to eliminate interference from dissolved oxygen and residual air. Upon initiating linear sweep voltammetry at 600 s, a pronounced increase in signal intensity at m/z = 32 was detected by DEMS, corresponding to the applied potential. This directly confirms the generation of oxygen on the working electrode surface, unequivocally demonstrating the occurrence of the oxygen evolution reaction and thereby providing direct evidence for the catalytic process. To further evaluate the electrocatalytic performance, Fe_0.26_Co_0.74_OOH/NF with a lower Fe content and FeOOH/NF without Co were synthesized on nickel foam (NF) as control samples using the same procedure (Figures S8 and S9). The OER activity of these catalysts was investigated in 1.0 M KOH electrolyte. The small redox peaks observed between 1.25 and 1.40 V in the inset of Figure 4a could be ascribed to the Co^3+^/Co^4+^ transition [12]. Therefore, linear sweep voltammetry (LSV) was recorded in the reverse scan mode to avoid interference.

As shown in the iR-corrected polarization curves (Figure 4a), Fe_0.42_Co_0.58_OOH/NF exhibited the lowest overpotential of only 220 mV at 10 mA·cm^−2^, which was significantly lower than Fe_0.26_Co_0.74_OOH/NF (280 mV), FeOOH/NF (279 mV), bare NF (370 mV), and even IrO_2_/NF (291 mV) [24], demonstrating its outstanding OER activity. It is worth noting that FeOOH/NF performed reasonably well at low current densities but showed a rapid decline in catalytic activity under high currents, which is consistent with the poor conductivity of FeOOH at elevated currents [6].

The Tafel slope, derived from LSV curves (Figure 4b), was used to further investigate the reaction kinetics. Fe_0.42_Co_0.58_OOH/NF exhibited the lowest Tafel slope (31.9 mV·dec^−1^), indicating more favorable kinetics compared to other catalysts. The electrochemical double-layer capacitance (C_dl_) was extracted from CVs recorded at different scan rates (Figure S10), and the electrochemical surface area (ECSA) was calculated accordingly (Figure 4e). The ECSA of Fe_0.42_Co_0.58_OOH/NF reached as high as 89.5 cm^2^, which was significantly larger than Fe_0.26_Co_0.74_OOH/NF (47.75 cm^2^) and bare NF (27.75 cm^2^), suggesting the provision of more accessible active sites. Electrochemical impedance spectroscopy (EIS) was employed to evaluate the interfacial charge-transfer ability. The Nyquist plots revealed that Fe_0.42_Co_0.58_OOH/NF exhibited a much smaller charge-transfer resistance (R_ct_) compared to the control samples (Figure 4d), demonstrating its superior interfacial charge transport capability. Based on quantitative gas measurements, we calculated the turnover number (TON) of the as-prepared catalysts. As shown in Table S4, the Fe_0.42_Co_0.58_OOH/NF exhibited the highest TON value of 1.31, while the reference samples Fe_0.26_Co_0.74_OOH/NF and FeOOH/NF showed TON values of 1.14 and 1.05, respectively. All catalysts demonstrated TON values exceeding unity, confirming their intrinsic catalytic nature, with the TON trend aligning consistently with their electrochemical activity. Moreover, Table S4 compares the over-potentials and Tafel slopes of high-performance Co-based catalysts reported in previous studies, highlighting that Fe_0.42_Co_0.58_OOH/NF remains at the forefront.

The durability of the catalysts was further evaluated through long-term electrochemical testing. As shown in Figure 4c, Fe_0.42_Co_0.58_OOH/NF maintained stable activity after 1000 continuous CV cycles. Furthermore, chronopotentiometric testing at a high current density of 600 mA·cm^−2^ confirmed that Fe_0.42_Co_0.58_OOH/NF retained its excellent catalytic activity for over 12.6 h of continuous operation (Figure 4f). Taken together, these results demonstrate that Fe_0.42_Co_0.58_OOH/NF combines outstanding catalytic activity with robust structural stability.

### 2.4. Mechanistic Investigation of the OER

**Figure 5:**
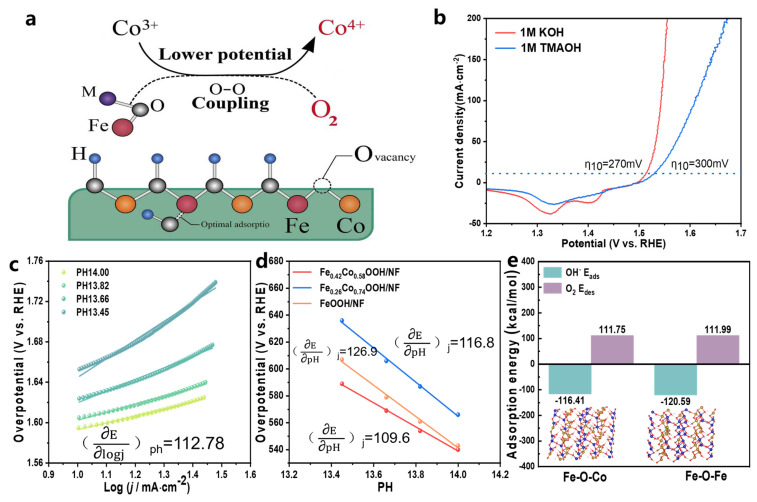
(**a**) Schematic diagram of Fe-M bonds promoting the OER mechanism; (**b**) LSV curves of Fe_0.42_Co_0.58_OOH/NF in 1M KOH and 1M TMAOH solution; (**c**) OER activities of Fe_0.42_Co_0.58_OOH/NF in KOH solutions with different pH values (14.00, 13.82, 13.66, and 13.45); (**d**) Fitting plot of the Overpotentials at 10 mA cm^−2^ vs. the pH value of the electrolytes; (**e**) Fe-O-Fe and Fe-O-Co sites’ adsorption energies and desorption energies of OH^−^ and O_2_.

The intrinsic relationship among the structural, chemical, and kinetic properties of Fe–M (M = Fe/Co) oxygen-bridged motifs was further examined to clarify the origin of the OER activity and catalytic mechanism of Fe_0.42_Co_0.58_OOH/NF (Figure 5a). In general, the OER can proceed through two possible pathways: the conventional adsorbate evolution mechanism (AEM) or the lattice oxygen oxidation mechanism (LOM). As shown in Figure S11, the LOM pathway involves O–O bond formation via the coupling of lattice oxygen with adsorbed oxygen species, with peroxide intermediates (O_2_^2−^) serving as characteristic markers. Tetramethylammonium cations (TMA^+^) are known to interact with O_2_^2−^ species and thereby suppress the LOM process [33]. Accordingly, tetramethylammonium hydroxide (TMAOH) was employed in this study as a chemical probe to discern the dominant reaction pathway. When OER was carried out in 1 M TMAOH electrolyte (Figure 5b), the catalytic activity of Fe_0.42_Co_0.58_OOH/NF decreased markedly compared with that in 1 M KOH, with the overpotential at 10 mA cm^−2^ (η_10_) increasing from 270 to 300 mV. This inhibition effect confirms that the OER on Fe_0.42_Co_0.58_OOH/NF proceeds primarily via the LOM pathway.

Further structural and defect-related evidence revealed the strong correlation between LOM promotion and Fe–M oxygen-bridged structures. Electron paramagnetic resonance (EPR) showed that the oxygen vacancy (V_o_) concentration in Fe_0.42_Co_0.58_OOH/NF was significantly higher than that of its precursor Fe_0.42_Co_0.58_-SO_4_/NF (Figure 3c). EXAFS fitting showed an increased Fe–O coordination number coupled with a decreased Fe–O–M (M = Fe/Co) coordination number, indicating partial deconstruction of the octahedral framework and the formation of additional boundary and unsaturated sites. This observation aligns with the EXAFS evidence that some di-μ-O bridges dissociate and convert into mono-μ-O bridges. As discussed in Section 3.2, di-μ-O motifs help preserve lattice stability, whereas mono-μ-O motifs are more prone to inducing oxygen vacancy formation and lattice oxygen activation, thereby triggering the LOM pathway. Thus, their dynamic inter-conversion is considered a critical factor that enables the catalyst to simultaneously maintain high stability and high activity under large current densities.

The structural evolution governs the resulting chemical properties, while changes in valence states and surface adsorption behaviors further support this correlation. Previous studies have shown that Fe-participated di-μ-O Fe–Co bridges play a crucial role in promoting the formation of high-valence Co species. Meanwhile, the in situ generated Co^4+^ species during the electrochemical process are widely recognized as the primary active centers [34]. The linear sweep voltammetry (LSV) curve exhibited a pronounced reduction peak in the range of 1.2–1.35 V, indicating the participation of Co^4+^-based reactive oxygen species (e.g., Co(VI), CoO_2_) (Figure 4a). Moreover, CV comparisons revealed that the Fe^3+^/Fe^4+^ transition potential of FeOOH/NF was around 1.45 V (Figure S12), consistent with previous literature [35], whereas in Fe_0.26_Co_0.74_OOH/NF and Fe_0.42_Co_0.58_OOH/NF, the Co^3+^/Co^4+^ transition potentials decreased to 1.05 V and 0.9 V, respectively, much lower than the reported value for pure CoOOH (≈1.25 V) [35]. In addition, the peak area of Fe_0.42_Co_0.58_OOH/NF increased markedly, indicating that Fe–Co interactions and the presence of (di-μ-O) Fe–Co bridges not only lower the Co^3+^/Co^4+^ transition potential but also enhance the generation of active Co^4+^ species. Nocera et al. proposed that the strong Lewis acidity of Fe^3+^ can reduce the energy barrier for the Ni^3+^/Ni^4+^ transition [36]; a similar effect has been observed in Co-based oxyhydroxides [12]. Therefore, Fe and Co act cooperatively through (di-μ-O) bridging to reduce the activation barrier for Co to reach higher oxidation states, thereby facilitating the generation of more Co^4+^ species at lower potentials. This accelerates the LOM pathway and enhances the overall OER kinetics.

At the adsorption level, density functional theory (DFT) calculations (Figure 5e, Figures S14 and S15) indicate that, under conditions where the O_2_ desorption energy is similar, OH^−^ preferentially adsorbs at the Fe^3+^–O–Fe^3+^ sites rather than the Fe–O–Co sites. This suggests that the Fe^3+^–O–Fe^3+^ oxygen bridge effectively adsorbs and stabilizes OH^−^, and its higher electron density and strong electron-accepting ability contribute to accelerating the oxygen evolution reaction (OER). This phenomenon further demonstrates that the Fe^3+^–O–Fe^3+^ oxygen bridge enhances the catalyst’s reaction activity by promoting the adsorption and activation of intermediates during the catalytic process.

From a kinetic standpoint, the pH-dependent activities of the three samples revealed that the overall catalytic performance increased with rising OH^−^ concentration within the pH range of 13.45–14.00 (Figure 5c and Figure S13). Combined with the current–pH response and Tafel slope analysis, the apparent reaction orders of FeOOH/NF and Fe_0.26_Co_0.74_OOH/NF were determined to be 0.89 and 0.86, respectively (Figure 5d). These values indicate that their catalytic activities are relatively insensitive to OH^−^ concentration and tend toward lattice oxygen depletion during the reaction. In contrast, Fe_0.42_Co_0.58_OOH/NF exhibited an apparent reaction order close to 1, demonstrating an approximately first-order dependence of current on OH^−^ concentration. This behavior suggests that lattice oxygen consumption was efficiently compensated by OH^−^ replenishment from the electrolyte, thereby enhancing its kinetic stability. Overall, these findings highlight that an optimized Fe/Co ratio can effectively regulate reaction kinetics, achieving a balance between catalytic activity and stability through pronounced synergistic effects.

In summary, Fe–M oxygen-bridged motifs play a pivotal role in bond formation and the rate-determining step by modulating defect states and the local electronic/valence environment, thereby synergistically optimizing the adsorption of *OH intermediates. This structural tuning lowers the energy barrier of the rate-limiting step and enhances apparent kinetics, all while preserving lattice stability. Consequently, Fe–M oxygen bridges serve as the core contributors to the exceptional OER performance of this system.

## 3. Experimental Section

### 3.1. Materials and Chemicals

The reagents and materials used in this study include: potassium hydroxide (KOH, analytical grade, purity 95%, Macklin Reagent Co., Ltd., Shanghai, China); cobalt nitrate hexahydrate (Co(NO_3_)_2_·6H_2_O, reagent grade, purity 99%, Macklin Reagent Co., Ltd. Shanghai, China); ferrous sulfate heptahydrate (FeSO_4_·7H_2_O, analytical grade, Shanghai Hushi Reagent Co., Ltd., Shanghai, China); tetramethylammonium hydroxide pentahydrate (TMAOH·5H_2_O, purity 97%, Aladdin Reagent Co., Ltd., Shanghai, China); anhydrous sodium sulfate (Na_2_SO_4_, analytical grade, Shanghai Hushi Reagent Co., Ltd., Shanghai, China); nickel foam (pore density 95 ppi, Kunshan Xingzhenghong Electronic Materials Co., Ltd., Kunshan, China). All chemicals were used as received without further purification. Ultrapure water (18.2 MΩ·cm) was used in all experiments.

### 3.2. Catalyst Preparation

#### 3.2.1. Synthesis of Fe_0.42_Co_0.58_OOH/NF

**Scheme 1:**
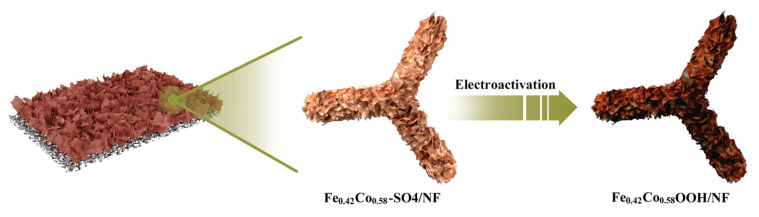
Formation process of Fe_0.42_Co_0.58_OOH/NF.

As shown in Scheme 1, first, 5.093 g of Co(NO_3_)_2_·6H_2_O was dissolved in 60 mL of isopropanol to form a homogeneous solution; then 0.4875 g of FeSO_4_·7H_2_O was dissolved in 20 mL of deionized water. Under vigorous stirring, the iron salt solution was slowly dropped into the cobalt salt solution, generating a stable nanoparticle dispersion. Pre-cleaned nickel foam substrates (3 × 3 cm^2^, treated by ultrasonic cleaning in HCl–ethanol solution and dried) were immersed completely into the mixed solution and left at room temperature for 24 h. The obtained product was thoroughly rinsed with deionized water, followed by drying in a vacuum oven at 30 °C for 12 h, affording the precursor Fe_0.42_Co_0.58_-SO_4_/NF. Electrochemical activation was performed in 1 M KOH electrolyte by subjecting the precursor to 10 cyclic voltammetry (CV) scans within 0.87–1.27 V (vs. RHE), using a graphite rod as the counter electrode, ultimately yielding the target catalyst Fe_0.42_Co_0.58_OOH/NF.

#### 3.2.2. Synthesis of Fe_0.26_Co_0.74_OOH/NF

The synthesis procedure was essentially identical to that of Fe_0.26_Co_0.74_OOH/NF, except for the precursor composition: 10.053 g of Co(NO_3_)_2_·6H_2_O was dissolved in 60 mL of isopropanol, while the Fe precursor remained unchanged at 0.4875 g of FeSO_4_·7H_2_O in 20 mL of water. The subsequent mixing, substrate immersion, washing, drying, and electrochemical activation steps were performed in the same way, yielding Fe_0.26_Co_0.74_OOH/NF.

#### 3.2.3. Synthesis of FeOOH/NF

In this case, the synthesis followed the same procedure as Fe_0.42_Co_0.58_OOH/NF, except that 5.093 g of Co(NO_3_)_2_·6H_2_O was replaced with 1.487 g of NaNO_3_ (molar equivalent to Co(NO_3_)_2_·6H_2_O), dissolved in 60 mL of isopropanol. Meanwhile, 0.4875 g of FeSO_4_·7H_2_O was dissolved in 20 mL of water and slowly added dropwise under stirring to form a uniform mixture. The pre-treated nickel foam substrate was immersed, allowed to react at room temperature, rinsed, dried, and then subjected to identical CV activation in 1 M KOH to yield FeOOH/NF.

### 3.3. Catalyst Characterization

The morphology of the catalysts was examined using field-emission scanning electron microscopy (FE-SEM, JSM-6330F, ZESSI sigma300, Jena, Germany) and transmission electron microscopy (TEM, JEM-2010HR, JEOL, Tokyo, Japan). The crystallographic structure of the materials was analyzed by X-ray diffraction (XRD, Rigaku D/max 2500/PC, smartlabSE, Tokyo, Japan), while local structural information was obtained from Raman spectroscopy (Renishaw, inVia, Thermo Scientific, Waltham, MA, USA). The chemical states of elements were determined by X-ray photoelectron spectroscopy (XPS, ESCA KAB 250, Thermo Fisher Scientific, K-Alpha, Waltham, MA, USA), with the C 1s peak calibrated to 284.8 eV. The metal loading and elemental ratios were quantified by inductively coupled plasma atomic emission spectroscopy (ICP-AES, TJA IRIS (HR), Agilent 720ES, Santa Clara, CA, USA). X-ray absorption fine structure (XAFS) measurements were performed at the 1W1B beamline of the Singapore Synchrotron Light Source (SSLS).

### 3.4. Electrochemical Measurements

Electrochemical measurements were carried out on a DH7003 electrochemical workstation (Donghua Instruments, Jingjiang, China). A graphite rod and a Hg/HgO electrode were used as the counter and reference electrodes, respectively. All potentials were converted to the reversible hydrogen electrode (RHE) scale according to the following equation:

E_RHE_ = E_Hg/HgO_ + 0.098 + 0.059 × pH

Prior to measurements, the electrolyte was saturated with O_2_, and all electrochemical data were corrected for 90%iR compensation. The ECSA could be estimated by measuring the non-Faradaic capacitive current associated with double-layer charging from the scan-rate dependence of cyclic voltammetry (CV), according to below Equation.

$$ECSA=\frac{C_{dl}}{C_{s}}$$

where C_dl_ is the double-layer capacitance, and C_s_ is the specific capacitance of any investigated electrode material. Here we used general specific capacities of C_s_ = 0.040 mF·cm^−2^ in 1 M KOH for estimating our surface area. C_dl_ could be extracted from recording CVs at various scan rates. In specific, the potential range of the CV measurements was selected between 0.60 and 0.70 V (versus RHE), in order to avoid the Faradaic and redox processes taking place. Also, the scan rates varied from 0.02, 0.04, 0.06, 0.08, 0.1 mV·s^−1^. These tests were all conducted in a static electrolyte. Here, the slope of the resulting charging current vs. scan rate plot was approximately regarded as C_dl_.

### 3.5. pH-Dependent Electrochemical Measurements

To investigate the effect of alkalinity on catalytic behavior, linear sweep voltammetry (LSV) was performed in KOH electrolytes with concentrations ranging from 0.1 to 5.0 M. The polarization curves obtained at different pH values were analyzed in the kinetic region, where the E–log j plots were linearly fitted to extract the corresponding Tafel slopes. The current densities at fixed potentials were further plotted as log j versus pH, and the apparent reaction order with respect to OH^−^ concentration was calculated using the Tafel slope, thereby quantitatively describing the dependence of current on OH^−^ activity.

### 3.6. TurnOver Number (TON)

The intrinsic activity of the catalyst was evaluated through TON calculation by performing chronopotentiometry at a constant current density of 600 mA·cm^−2^ for 60 s in a sealed two-compartment electrochemical cell, where the evolved gases were collected via a water-filled syringe connected to the anode outlet. The oxygen volume was determined from the total collected gas volume based on the water electrolysis stoichiometric ratio (H_2_:O_2_ = 2:1), and the moles of oxygen produced were calculated using the ideal gas law.

TON was then obtained using the equation:

TON = (n_O2_ × NA)/n_metal_

where n_O2_ is the moles of oxygen produced, NA is Avogadro’s constant (6.022 × 10^23^ mol^−1^), and n_metal_ represents the total moles of metal atoms (Co + Fe) on the working electrode, determined from the catalyst loading mass (2.83 mg) and its chemical composition.

### 3.7. Computational Methods

All quantum chemical calculations were performed using the CP2K v2024.3 package, with geometric optimization and energy calculations conducted via the xTB-GFN1 method under the Quickstep module. The Ewald summation was enabled to handle the electrostatic interactions of the periodic system; the net charge of the system was set to 0 and the spin multiplicity to 1. Meanwhile, geometric structure minimization optimization was carried out using the BFGS optimizer (with a trust radius of 0.2 Å and a maximum number of iterations of 500), and the convergence criteria were defined as follows: maximum atomic displacement ≤ 3.0 × 10^−3^ Å, root-mean-square (RMS) atomic displacement ≤ 1.5 × 10^−3^ Å, maximum force ≤ 4.5 × 10^−4^ a.u., and RMS force ≤ 3.0 × 10^−4^ a.u. Additionally, fixed constraints were applied to the XYZ Cartesian coordinates of some atoms. For the self-consistent field (SCF) calculations, the orbital transformation (OT) algorithm was adopted, with the maximum number of inner loop steps set to 25 and the convergence threshold to 1.0 × 10^−6^, as well as the maximum number of outer loop steps set to 20 and the convergence threshold to 1.0 × 10^−6^. Structural trajectories were output in XYZ format for subsequent analysis.

## 4. Conclusions

This study highlights the pivotal role of Fe–M oxygen-bridged motifs in the OER process. During the electrochemical self-reconstruction process, dynamic interconversion between mono-μ-O and di-μ-O bridging units occurs, which not only provides abundant active sites but also preserves the structural stability. In addition, Fe^3+^–O–Fe^3+^ motifs generated under the Fe-rich environment act synergistically with high-valence Co species to promote the LOM, thereby accelerating the overall reaction kinetics. Our findings emphasize the importance of oxygen bridges in the synergistic regulation of catalytic activity and stability, providing structural insights for the rational design of Fe-rich Fe–Co-based catalysts. Future studies combining multi-scale simulations with advanced in situ characterization are expected to further elucidate the evolution of oxygen-bridged structures and explore their stability mechanisms under long-term operation and large-scale applications.

## Data Availability

The original contributions presented in this study are included in the article/Supplementary Materials. Further inquiries can be directed to the corresponding authors. This study does not involve humans, and thus no informed consent from subjects is required.

## Supplementary Materials

The following supporting information can be downloaded at: https://www.mdpi.com/article/10.3390/molecules31010096/s1, Figure S1: Fe_0.42_Co_0.58_-SO_4_/NF SEM. Figure S2: High-Resolution Lattice Spacing Distribution of Fe0.42Co0.58-SO4/NF. Figure S3: XRD pattern of Fe_0.42_Co_0.58_-SO_4_/NF. Figure S4: The XPS spectra of Co 2p of Fe_0.42_Co_0.58_-SO_4_/NF. Figure S5: The SEM of Fe_0.42_Co_0.58_OOH/NF. Figure S6: K-space (photoelectron wave vector space). Figure S7. In situ DEMS monitoring of the oxygen evolution reaction over the Fe0.42Co0.58OOH/NF catalyst (*m*/*z* = 32). Figure S8: Fe_0.26_Co_0.74_OOH/NF SEM. Figure S9: FeOOH/NF SEM. Figure S10: CVs at various scan rates Figure S11: Schematic illustration of different reaction pathways for the oxygen evolution reaction (LOM and AEM). Figure S12: CV tests of different catalysts in the redox potential region. Figure S13: (a) Fe_0.26_Co_0.74_OOH (b)FeOOH pH-Dependent test. Figure S14: Adsorption energy of OH^−^ intermediate at different sites (a) Fe-O-Co(b) Fe-O-Fe. Figure S15: Adsorption energy of O_2_ at different sites (a) Fe-O-Co(b) Fe-O-Fe. Table S1: Elemental Composition of Sample Fe_0.42_Co_0.58_-SO_4_/NF (SEM Mapping). Table S2: Elemental Composition of Sample Fe_0.42_Co_0.58_OOH/NF (SEM Mapping) Table S3: EXAFS fitting parameters at the Fe K-edge various samples (S02 = 0.70). Table S4. Turnover number (TON) calculations of the catalysts based on quantitative gas measurements. Table S5: Comparison of OER activities of other catalysts in KOH solution. References [37,38,39,40,41,42,43,44,45,46] are cited in the Supplementary Materials.
