# Supplementary material for: Oxygen Bridge Governs OER via Deep Self-Reconstruction in Fe–Co Oxyhydroxides
Source: Molecules. 2025 Dec 25;31(1):96. doi: 10.3390/molecules31010096 (PMC12786700; doi:10.3390/molecules31010096)
Supplement: Supplementary file 1 [file molecules-31-00096-s001.zip › molecules-3985592-supplementary.pdf]

Oxygen Bridge Governs OER via Deep Self-Reconstruction in Fe–Co Oxyhydroxides

Mingyu Liu [1], Bowen Pei [1], Hongyu Ba [1], Wei Ni [1], Huaheng Zhao [1], Shuang Chen [1,*], Jiamin Zhao [2,*] and Jinsheng Zhao [2,*]

[1] College of Chemistry and Chemical Engineering, China University of Petroleum (East China), Qingdao 266580, China; liumingyu991204@163.com (M.L.); peibowen2024@163.com (B.P.); 17669793559@163.com (H.B.); nw17585134644@163.com (W.N.); 15212531621@163.com (H.Z.)

[2] School of Chemistry and Chemical Engineering, Liaocheng University, Liaocheng 252059, China

* Correspondence: chsh1030@163.com (S.C.); zhaojiamin08031223@163.com (J.Z.); j.s.zhao@163.com (J.Z.)

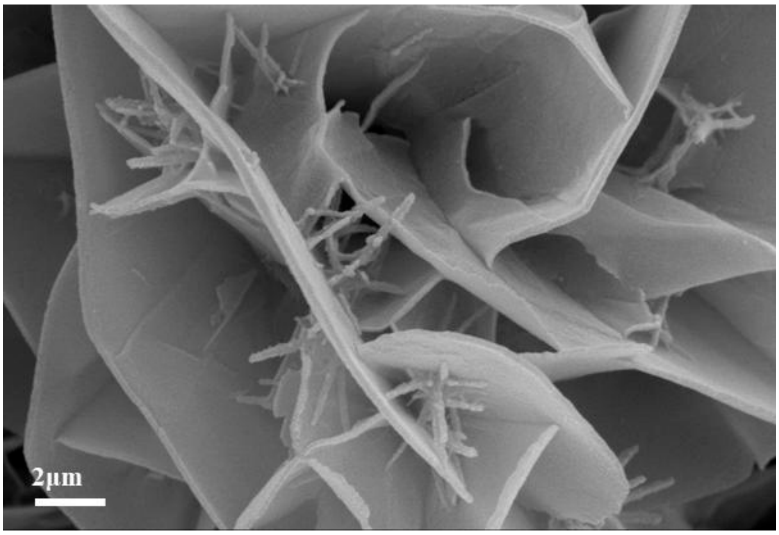

Figure S1. $Fe_{0.42}Co_{0.58}$-SO_4/NF SEM.

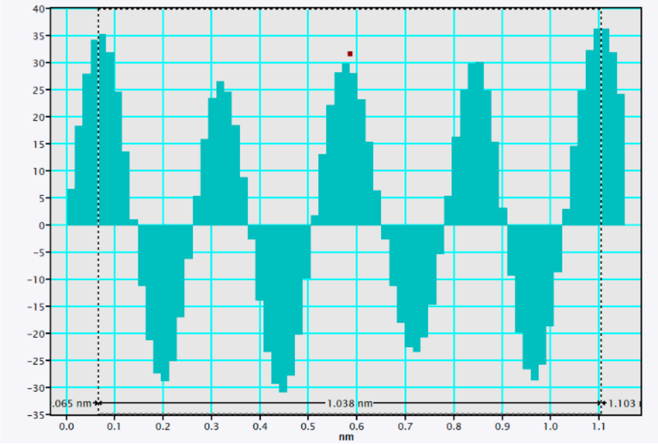

Figure S2. High-Resolution Lattice Spacing Distribution of $Fe_{0.42}Co_{0.58}$-SO_4/NF.

Table S1. Elemental Composition of Sample $Fe_{0.42}Co_{0.58}$-SO_4/NF (SEM Mapping).

Samples	Wt%
Fe	23.89
Co	23.02
O	41.14
S	4.37
Others	7.57

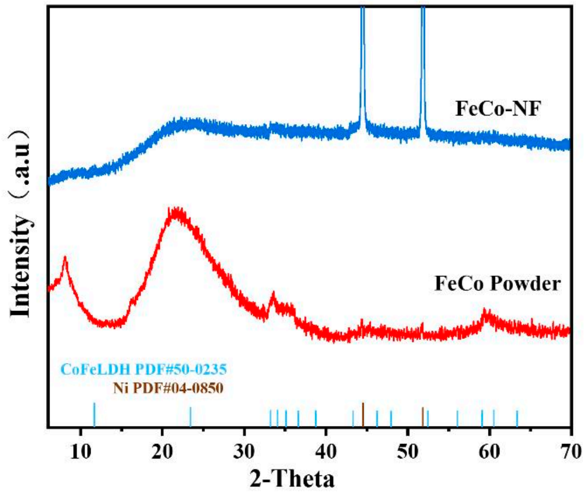

Figure S3. XRD pattern of $Fe_{0.42}Co_{0.58}$-SO_4/NF.

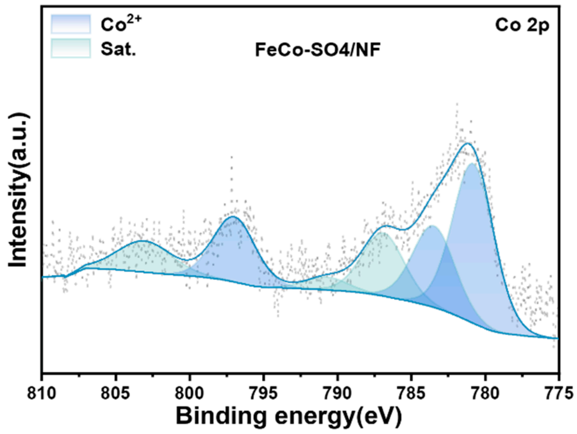

Figure S4.The XPS spectra of Co 2p of $Fe_{0.42}Co_{0.58}$-SO_4/NF .

Table S2. Elemental Composition of Sample $Fe_{0.42}Co_{0.58}OOH$/NF (SEM Mapping)

Samples	Wt%
Fe	36.79

Co	39.70
O	23.45
S	0.06

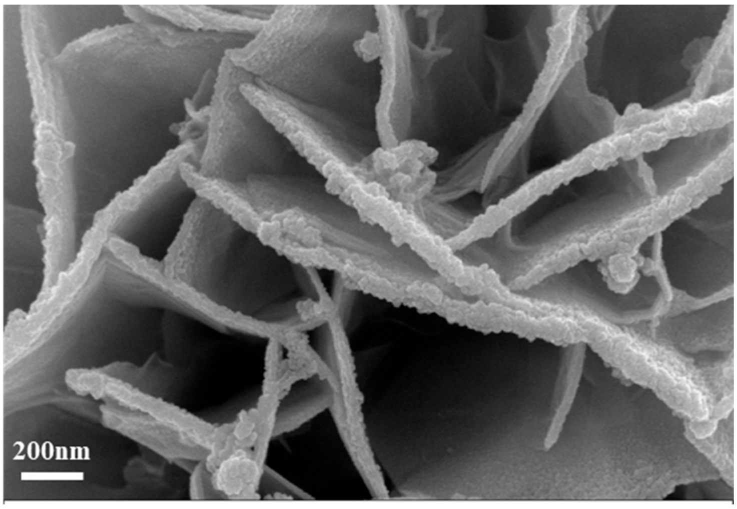

Figure S5. The SEM of $Fe_{0.42}Co_{0.58}OOH/NF$

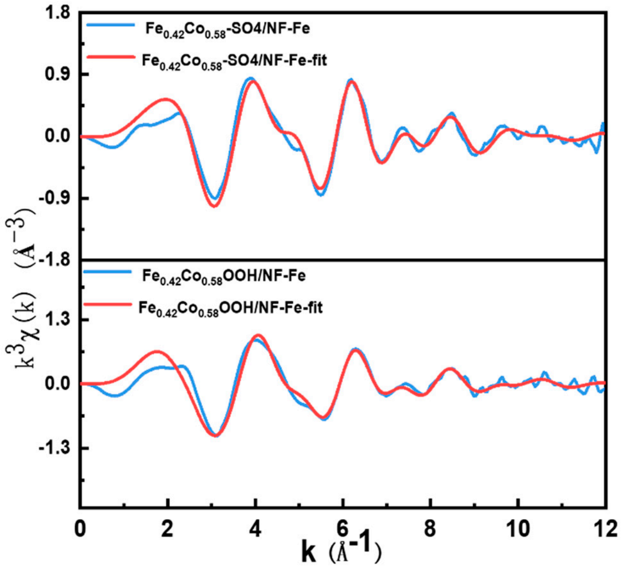

Figure S6. K-space (photoelectron wave vector space).

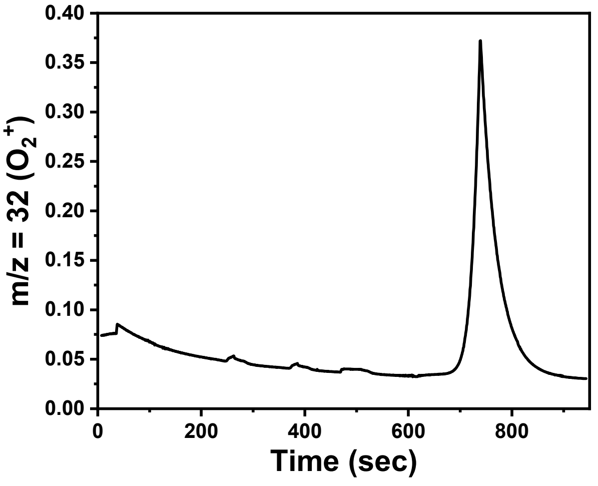

Figure S7. In situ DEMS monitoring of the oxygen evolution reaction over the $Fe_{0.42}Co_{0.58}OOH$/NF catalyst (m/z = 32).

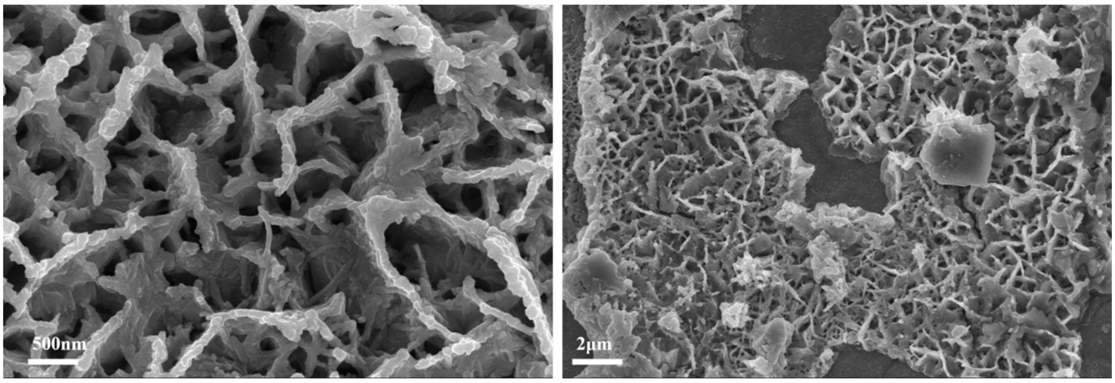

Figure S8. $Fe_{0.26}Co_{0.74}OOH$/NF SEM.

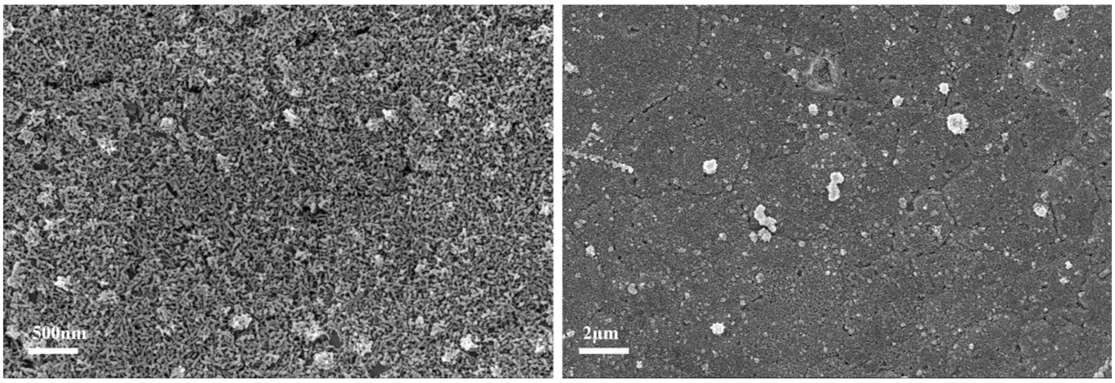

Figure S9. FeOOH/NF SEM.

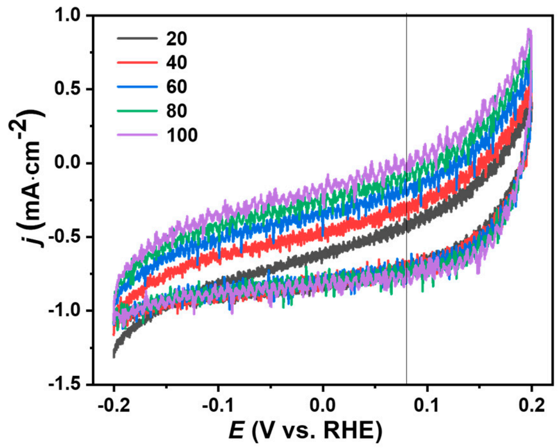

Figure S10. CVs at various scan rates

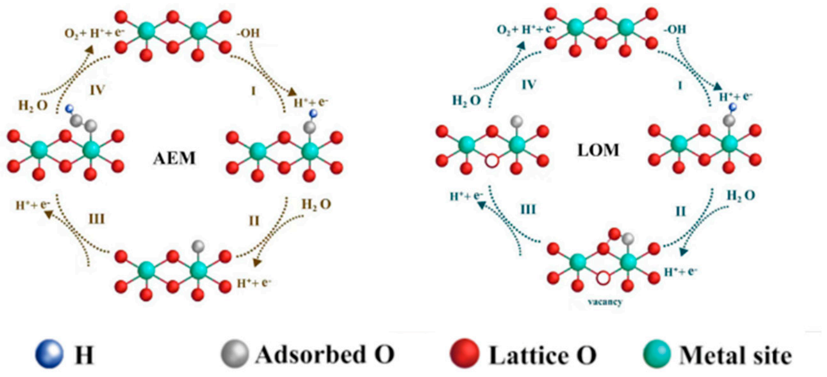

Figure S11. Schematic illustration of different reaction pathways for the oxygen evolution reaction (LOM and AEM).

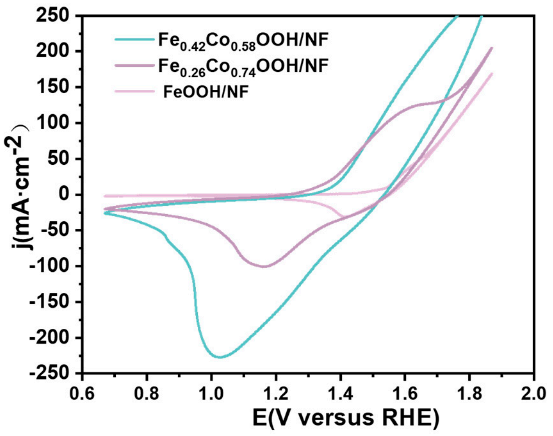

Figure S12. CV tests of different catalysts in the redox potential region.

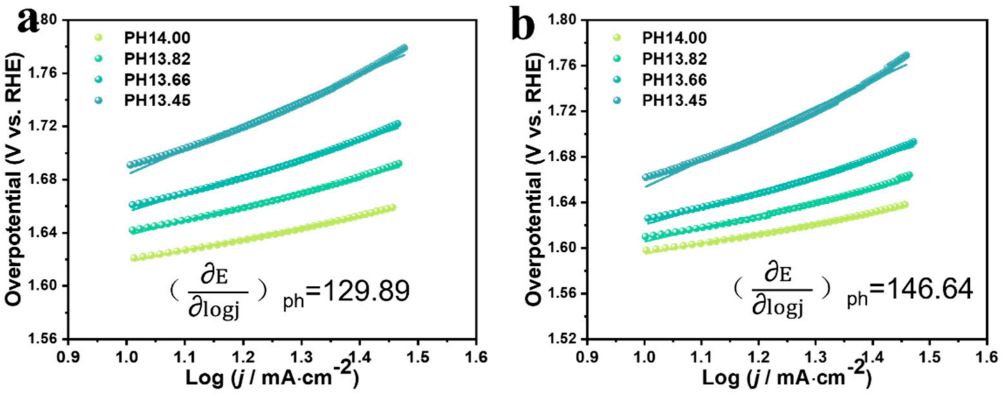

Figure S13. (a) $Fe_{0.26}Co_{0.74}OOH$ (b)FeOOH pH-Dependent test.

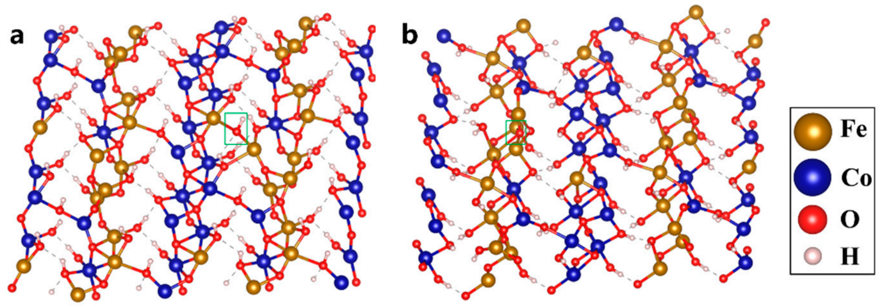

Figure S14. Adsorption energy of OH^- intermediate at different sites (a) Fe-O-Co(b) Fe-O-Fe.

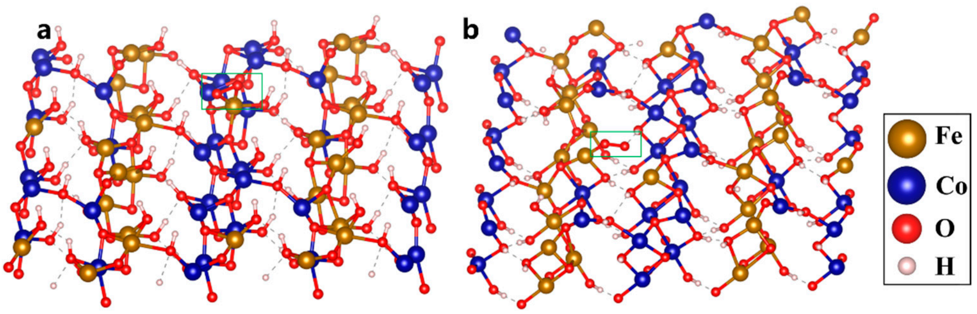

Figure S15. Adsorption energy of O_2 at different sites (a) Fe-O-Co(b) Fe-O-Fe.

Table S3. EXAFS fitting parameters at the Fe K-edge various samples (S02=0.70);

[a] *CN*: average coordination numbers; [b] *R*: bond distance; [c] σ^2: Debye-Waller factors; [d] ΔE_0: the inner potential correction. *R* factor: goodness of fit. *S*02 was set as 0.85/0.805 for Co-O/Co-Co , which was obtained from the experimental EXAFS fit of reference CoO by fixing CN as the known crystallographic value and was fixed to all the samples.

samples	path	C. N.[a]	R (Å) [b]	σ^2 ($\times 10^{-3}$ Å^2) [c]	ΔE (eV) [d]	R factor[e]
Fe-1	Fe-O	4.7±0.6	2.00±0.01	8.9±2.3	-0.4±1.1	0.011
	Fe-O-M	6.5±2.0	3.06±0.02	7.4±1.8		
Fe-2	Fe-O	5.5±0.6	1.96±0.01	4.9±0.8	-3.7±1.1	0.005
	Fe-O-M	4.7±2.1	3.25±0.02	6.6±2.5		

Table S4. Turnover number (TON) calculations of the catalysts based on quantitative gas measurements.

Sample	Total Gas Volume (mL)	O_2 Volume (mL)	Total O_2 Molecules	Total Metal Atoms	TON
$Fe_{0.42}Co_{0.58}OOH$/NF	3.0	1.0	2.46*10^{19}	1.88*10^{19}	1.31
$Fe_{0.26}Co_{0.74}OOH$/NF	2.6	0.87	2.14*10^{19}	1.88*10^{19}	1.14
FeOOH/NF	2.4	0.8	1.97*10^{19}	1.88*10^{19}	1.05

Table S5. Comparison of OER activities of other catalysts in KOH solution.

Electrocatalysts	$\eta_{10\ mA\ cm^{-2}}$/ mV	Tafel slope/ mV dec^{-1}	References
$Fe_{0.42}Co_{0.58}OOH$/NF	220	31.9	This work
CoFe-PBA	305	36.1	*1*
Fe-CoF 2-300	230	41.9	*2*
Q-LDH-0.1	270	66	*3*
CoFeO @ BP	266	42	*4*
CFS-2，Co： Fe = 1：1	291	65	*5*
Fc-CoxSy	304	54.2	*6*
FeCoOOH-Pd	265	37.5	*7*
FeCoS y /NCDs-3	284	52.1	*8*
FeNi LDH/MOF	255	24	*9*
S–$Ni_{4/5}Fe_{1/5}$-LDHs	257	61.5	*10*

References

1 Diao, F.; Kraglund, M. R.; Cao, H.; Yan, X.; Liu, P.; Engelbrekt, C.; Xiao, X. Moderate heat treatment of CoFe Prussian blue analogues for enhanced oxygen evolution reaction performance. *Journal of Energy Chemistry.* **2023**, *78*, 476–486.

2 Li, M.; Gu, Y.; Chang, Y.; Gu, X.; Tian, J.; Wu, X.; Feng, L. Iron doped cobalt fluoride derived from CoFe layered double hydroxide for efficient oxygen evolution reaction. *Chemical Engineering Journal.* **2021**, *425*, 130686.

3 Yao, R.; Wu, J.; Kansara, S.; Sun, Z.; Kang, H.; Liu, F.; Wang, K.; Hu, J.; Li, X.; Wu, D.; Hwang, J.-Y.; Xiong, S. Recycling defunct lithium-ion battery cathodes to quaternary layered double hydroxides for efficient oxygen evolution reaction. *Advanced Science.* **2025**, *12*(26), e2501957.

4 Li, X.; Xiao, L.; Zhou, L.; Xu, Q.; Weng, J.; Xu, J.; Liu, B. Adaptive bifunctional electrocatalyst of amorphous CoFe oxide@2D black phosphorus for overall water splitting. *Angewandte Chemie International Edition.* **2020**, *132*(47), 21292–21299.

5 Li, J.; Liu, Z.; Li, W.; Ma, H.; Fang, P.; Xiong, R.; Pan, C.; Wei, J. In situ interfacial engineering of $MnIn_2S_4@In_2S_3$ hollow nanotubes for enhanced photocatalytic production of H_2O_2 and antibiotic degradation. *Journal of Colloid and Interface Science.* **2025**, *682*, 1–10.
6 Thangasamy, P.; Oh, S.; Nam, S.; Randriamahazaka, H.; Oh, I. K. Ferrocene-incorporated cobalt sulfide nanoarchitecture for superior oxygen evolution reaction. *Small.* **2020**, *16*(31), 2001665.
7 Yang, S.; Lu, L.; Zhan, P.; Si, Z.; Chen, L.; Zhuang, Y.; Qin, P. Amorphous hetero-structure iron/cobalt oxyhydroxide with atomic dispersed palladium for oxygen evolution reaction. *Applied Catalysis B: Environment and Energy.* **2024**, *355*, 124213.
8 Wu, L.; Qin, H.; Ji, Z.; Zhou, H.; Shen, X.; Zhu, G.; Yuan, A. Nitrogen-Doped Carbon Dots Modified Fe–Co Sulfide Nanosheets as High-Efficiency Electrocatalysts toward Oxygen Evolution Reaction. *Small.* **2024**, *20*(4), 2305965.
9 Huo, J.; Wang, Y.; Yan, L.; Xue, Y.; Li, S.; Hu, M.; Jiang, Y.; Zhai, Q.-G. In situ semi transformation from heterometallic MOFs to Fe-Ni LDH/MOF hierarchical architectures for boosted oxygen evolution reaction. *Nanoscale.* **2020**, *12*(27), 14514-14523.
10 Li, S.; Liu, J.; Duan, S.; Wang, T.; Li, Q. Tuning the oxygen evolution electrocatalysis on NiFe-layered double hydroxides via sulfur doping. *Chinese Journal of Catalysis.* **2020**, *41*(5), 847–852.
